# Comparison of the Association of Excess Weight on Health Related Quality of Life of Women with Polycystic Ovary Syndrome: An Age- and BMI-Matched Case Control Study

**DOI:** 10.1371/journal.pone.0162911

**Published:** 2016-10-13

**Authors:** Farnaz Shishehgar, Fahimeh Ramezani Tehrani, Parvin Mirmiran, Sepideh Hajian, Ahmad Reza Baghestani

**Affiliations:** 1 Department of Midwifery and Reproductive Health, Faculty of Nursing and Midwifery, Shahid Beheshti University of Medical Sciences, Tehran, Iran; 2 Reproductive Endocrinology Research Center, Research Institute for Endocrine Sciences, Shahid Beheshti University of Medical Sciences, Tehran, Iran; 3 Nutrition and Endocrine Research Center, Research Institute for Endocrine Sciences, Shahid Beheshti University of Medical Sciences, Tehran, Iran; 4 Department of Biostatistics, School of Paramedical Sciences, Shahid Beheshti University of Medical Sciences, Tehran, Iran; Weill Cornell Medical College Qatar, QATAR

## Abstract

**Background:**

It is assumed that obesity adversely affects the health related quality of life (HRQOL) of women with polycystic ovary syndrome (PCOS), not only due to the excess weight, but also due to several other obesity induced metabolic and reproductive consequences. We aimed to compare the effects of excess body weight on the HRQOL between women with PCOS and controls.

**Methods:**

This is a case control study of 142 women with PCOS and 140 age- and BMI- matched controls. The Iranian version of short form health survey 36 (SF 36) was used to assess HRQOL. Domains of SF 36 were compared in women with PCOS and controls using multivariate analysis of covariance. The Pearson correlation was used to assess the correlation between body mass index (BMI) and domain scores of SF 36, and the differences between two correlations in cases and controls, using Fisher’s Z test.

**Results:**

Women with PCOS had significantly lower scores for both, the physical and the mental component summary scales, compared to controls. In the cases, a significant negative correlations were observed for BMI with physical function (r = - 0.301, P<0.001), bodily pain (r = - 0.23, P = 0.006), and physical summary score (r = -0.3, P = 0.007). In controls, significant correlation was seen for BMI with bodily pain (r = - 0.3, P<0.001) and physical summary score (r = - 0.27, P = 0.001). The differences between correlations of physical function with BMI in PCOS and controls were statistically significant (Z = -2.41, P = 0.008).

**Conclusion:**

Although the physical aspects of HRQOL are adversely affected by overweight in both PCOS and controls, these impaired effects are greater in women with PCOS.

## Introduction

Polycystic ovary syndrome is one of the most common endocrinopathies in reproductive aged women, with a prevalence ranging between 4–20% [1, 2]. The heterogeneous clinical manifestations of PCOS including menstrual and ovulatory dysfunction, infertility, hirsutism and increased cardiovascular risk factors, all have negative effects on health related quality of life (HRQOL) [3, 4, 5]. Although the impact of obesity on various aspects of quality of life of general populations is unclear, the physical component of HRQOL is decreased by obesity; associations between body mass index and the mental components of HRQOL vary [6], with either a decline [7] or sometimes even improvement [8]. Obesity is associated with PCOS and exacerbates its clinical features [9]; however there are inconsistent data regarding the association between obesity and quality of life of women with PCOS, with some studies reporting obesity as a main mediator in the association between PCOS and reductions in HRQOL [10], whereas others document no relationship between obesity and HRQOL [11].

It is assumed that obesity has adverse effects on the HRQOL of women with PCOS, not only due to excess weight, as obesity in these women may also adversely affect their reproduction resulting in anovulation, subfertility and irregular menses, consequences eventually influencing their HRQOL. Furthermore, obesity in women with PCOS is also associated with more negative metabolic consequences e.g. insulin resistance [12]; as a result their HRQOL may be additionally influenced by comorbidities of obesity. The impacts of excess weight on various domains of HRQOL of women were not similar; Benetti et al. (2015) [13] and Hollander et al. (2013)[14] reported that body weight was correlated to the worsening in all aspect of HRQOL, Kozak et al. (2011) [15] showed that obesity is adversely associated with physical but not mental HRQoL; these studies were mostly conducted in western countries and the impact of excess weight on HRQOL of women with PCOS has not been compared with their age and BMI matched counterparts.

Considering the severe adverse effects of excess body weight on HRQOL among individuals with metabolic disorders [16], and the impact of obesity on the reproductive characteristics of women with PCOS, we hypothesized that in women with PCOS the associations of excess weight on HRQOL differ from those in healthy controls, by comparing the effects of excess body weight on HRQOL between women with PCOS and their age and BMI matched counterparts.

## Materials and Methods

This prospective study was conducted from January to July 2014; the case group was recruited from among women with PCOS aged 18–40 years, attending the Reproductive Endocrinology Research Center of Shahid Beheshti University of Medical Sciences. PCOS was defined using the criteria of the Androgen Excess Society (AES) as the combination of hirsutism and/or hyperandrogenemia, oligo-anovulation and/or polycystic ovaries, after excluding other etiologies of androgen excess and an ovulatory infertility (hyperprolactinemia, thyroid dysfunction, non-classic 21-hydroxylase deficiency (NCCAH) and Cushing’s syndrome) [17]. Controls were selected from among women attending gynecologic centers affiliated to Shahid Beheshti University of Medical Sciences in Tehran for their annual gynecological exam (performing pap smear or Papanicolau test, a screening test to detect potentially pre-cancerous and cancerous processes in the cervix, none of these women had any specific complaint. We frequency-matched our control subjects with PCOS cases based on age and BMI levels. To do this, we first categorized our PCOS cases into nine age and BMI groups. Subjects were then subdivided into < 25, 25–30 and over 30 years old, and further into BMI <25, 25–30 and over 30 kg/m^2^ groups. We excluded pregnant and breastfeeding women, those using oral contraceptive or those receiving drugs for infertility, those with a history of chronic disease or cancer and those suffering from acute mental tension due to death of a family member or similar conditions; none of the women, PCOS or controls, had history of depression or anxiety. Calculation of sample size was based on preliminary study on 50 women with PCOS and controls. Considering α = 0.05, and power = 0.95, the mean and standard deviation difference between two groups (in all domains of SF-36) were computed and maximum sample size for this study was calculated, and the sample size for each group was hence estimated at least 126 people in each group. Finally of 150 women with PCOS recruited for this study, 6 women were not willing to participate and 2 cases used infertility medications and of controls, (n = 150), 7 women were not willing to participate and 3 had diabetes; as a result 282 women including 142 cases and 140 controls completed study.

All participants completed a comprehensive questionnaire which included socio-demographic information, reproductive and menstrual history. The Iranian version of the short form health survey 36 (SF 36), used for assessing HRQOL, included eight domains; physical functioning (PF), role limitation due to physical problem (RP), bodily pain (BP), general health perception (GH), vitality (VT), social functioning (SF), role limitation due to emotional problem (RE) and mental health (MH). These eight scales are pooled into two summary measures the physical (PCS) and mental component summary scales (MCS), dimensions which are scored from 0 (minimum of HRQOL) to 100 (no defect) [18]. Validity and reliability of SF36 for Iranian populations have been confirmed, with convergent validity ranging from 0.58 to 0.95 and Cronbach's alpha coefficients ranging from 0.77 to 0.90 (alpha = 0.65) [19]. All participants underwent clinical examinations to document body weight, height, waist (WC), hip circumferences (HC). Body mass index (BMI) was measured as weight in kilograms, divided by the height in meters squared (kg/m²). BMI levels were classified as follows: BMI 18–24.9 kg/m² as normal, and BMI ≥ 25 kg/m² as overweight or obese. Hirsutism was assessed using the modified Ferriman-Gallwey scoring method [20], by the main investigator (F.S) under supervision of a gynecologist (F.R.T). This study was approved by the ethical committee of the Research Institute for Endocrine Sciences (RIES) and written informed consent was obtained from all participants.

## Statistical Analysis

Continuous variables, checked for normality, using the one-sample Kolmogorov-Smirnoff test, are expressed as mean ± standard deviation and log transformation was used for variables, not normally distributed. Categorical variables are expressed as percentages and the Chi–square test was utilized to compare differences in categorical variables among the study groups. A multivariate analysis of variances (MANOVA) was conducted to determine the differences in the SF-36 measures among each demographic group. Univariate analyses and post hoc tests were performed following significant multivariate effects.

Multivariate analyses of covariance (MANCOVAs) were utilized to examine differences in multidimensional scales of the SF- 36, with study groups as the between-group factor and BMI groups, age, parity, FG scores and menstrual regularity as covariates. Significant multivariate effects were followed up with the Bonferroni-adjusted univariate ANOVA. After adjustment for confounders (age, parity, FG scores, period regularity) the relationship between BMI and domains score of SF36 was examined using the Pearson correlation test. Fisher’s Z test was utilized to ascertain whether the correlation between BMI and domains of HRQOL is the same in both women with and those without PCOS. Data analysis was performed using the SPSS 20.0 PC package (SPSS Inc., Chicago, IL); p values < 0.05 were considered statistically significant.

## Results

Characteristics of women with PCOS and controls are illustrated in Table 1. Of 142 women with PCOS, 38% were hirsute.

**Table 1:** Characteristics of participants according to the study groups; women with polycystic ovary syndrome (PCOS) and controls.

Characteristics	PCOS(n = 142)	Control(n = 140)	P-value
**Age (years)**	28.6 ± 4.9	29.0 ± 5.8	0.631
**Education**			
Less than Diploma	22 (15.5%)	33 (23.6%)	0.087
Diploma & higher	120 (84.5%)	107 (76.4%)	
**Employment**			
Unemployed	96 (67.6%)	101(72.1%)	0.406
Employed	46 (32.4%)	39 (27.9%)	
**Marital status**			
Single	32 (22.5%)	33 (23.6%)	0.836
Married	110 (77.5%)	107 (76.4%)	
**Parity**			
Nulliparous	106 (74.6%)	69 (49.3%)	<0.001
Parity ≥ 1	36 (25.4%)	71 (50.7%)	
**Weight (kg)**	69.4 ± 15.0	67.5 ± 12.9	0.048
**BMI (kg/m**^**2**^**)**	26.6 ± 5.7	26.0 ± 4.8	0.133
**Waist circumference (cm)**	85.3 ± 14.0	85.2 ± 12.9	0.893
**Ferriman-Gallwey scores of hirsutism**	6 (3–10)	0 (0–1)	<0.001
**Period regularity**			
Yes	23 (%16.2)	140 (%100)	NA
No	119 (%83.8)	-	

Quantitative variables are presented as mean ± SD; categorical variables are expressed as percentage. NA; not applicable

Table 2 illustrates the effects of influencing factors on domains of health related quality of life of women with PCOS and controls; the multivariate analysis for PCOS status showed wilks λ = 0.035, F = 4.64 and P<0.001; age, marital status, educational levels, BMI and parity had statistically significant effects on the health related quality of life of controls. The effects of age, BMI and hirsutism on health related quality of life of women with PCOS were also statistically significant.

**Table 2:** Factors influencing sub scores of Health Related Quality Of Life (HRQOL) and summary scores of HRQOL (mental and physical) of women with polycystic ovary syndrome and controls.

**Multivariate analysis of variance of factors on 8 domain scales scores of HRQOL**						
	**PCOS (n = 142)**			**Controls (n = 140)**		
Factors	Wilks’s lambda value	F	p-value	Wilks’s lambda value	F	p-value
Age	0.884	2.17	0.033	0.799	4.11	<0.001
Marital status	0.90	1.77	0.087	0.811	3.81	<0.001
Educational levels	0.97	0.513	0.845	0.851	2.87	0.006
Employment Status	0.96	0.558	0.81	0.928	1.27	0.261
BMI	0.81	3.71	<0.001	0.77	4.77	<0.001
Parity	0.89	2	0.06	0.86	2.53	0.013
Period regularity	0.93	1.13	0.34			
Hirsutism (FG scores)	0.8	4.16	0.001			
Multivariate analysis of variance of factors on Summary scores (PCS, MCS) of HRQOL						
	**PCOS (n = 142)**			**Controls (n = 140)**		
Factors	Wilks’s lambda value	F	p-value	Wilks’s lambda value	F	p-value
Age	0.96	2.3	0.1	0.88	8.66	<0.001
Marital status	0.96	2.24	0.11	0.88	9.01	<0.001
Educational levels	0.99	0.517	0.59	0.95	3.22	0.043
Employment Status	0.99	0.216	0.8	0.95	2.99	0.053
BMI	0.92	5.2	0.007	0.94	4	0.02
Parity	0.97	2.11	0.12	0.9	7.55	0.001
Menstrual regularity	0.99	0.4	0.66			
Hirsutism	0.91	0.65	0.002			

Table 3 compares the mean scores of health related quality of life’s domains of women with and without PCOS; women with PCOS had significantly lower VT, MH, SF, BP, GH, PCS and MCS scores than controls.

**Table 3:** Comparisons of mean scores of health related quality of life’s domains of women with polycystic ovary syndrome with controls.

Domains of HRQOL	PCOS(n = 142)	Controls(n = 140)	MANOVA P-value	Wilks' Lambda P-value
	Mean± SD	Mean± SD		**<0.001**
Vitality	52.18±1.26	64.6±1.67	<0.001	
Mental health	54.99±1.93	68.82±1.94	<0.001	
Role limitation due to emotional problems	66.84±2.82	71.27±2.84	0.27	
Social Functioning	70.89±2.11	79.45±2.12	0.005	
Bodily pain	70.89±2.12	77.48±2.13	0.019	
General health perception	63.04±1.55	68.72±1.56	0.011	
Physical Functioning	80.29±1.89	82.35±1.9	0.445	
Role limitation due to physical problems	71.66±2.82	76.24±2.76	0.241	
Physical component summary scale	71.35±1.43	76.2±1.44	0.018	**<0.001**
Mental component summary scale	61.23±1.49	71.03±1.5	0.001	

Table 4 compares the mean scores of the domains of health related quality of women with and without PCOS in various demographic subgroups. Univariate analysis showed that, compared to older women, those aged <30 years, had significantly higher PF and lower MH in cases and higher PF and BP in controls (P<0.05) (Table 4). In controls, the PCS for the younger age group (< 30 years) was significantly higher than those for the older age women (P = 0.002). Regarding marital status, univariate analysis showed that unmarried controls had significantly higher PF, RP, BP, SF and PCS scores than married ones (P<0.05). For educational levels, the univariate analysis showed significant differences for BP in non-PCOS women; less educated women had significantly lower BP score of SF 36 than highly educated women (66.22±3.84 vs. 80.95±2.3 F = 2.87 P = 0.006). Healthy women with higher education had significantly higher PCS scores than less educated ones (77.67±1.37 vs. 71.43±2.47, P = 0.029) (data in S3 Table). For hirsutism, the univariate analysis showed significant differences for mental aspects of SF 36. In women with PCOS; non hirsute women with PCOS had significantly higher scores in VT, SF, MH, MCS, than hirsute women with PCOS (Table 4).

**Table 4:** Effect of various characteristics on HRQOL in women with and without PCOS.

**HRQOL domains**	**Sub-groups (Age)**		**Tests**	
	**< 30 years (n = 79)**	**≥30 years (n = 63)**	*MANOVA P-value	**Wilks' Lambda P-value
**PCOS**				**0.033**
Physical Functioning	84.61±2.66	74.88±2.98	0.016	
Mental health	50.48±2.82	60.65±3.16	0.018	
**Controls**	**< 30 years (n = 76)**	**≥30 years (n = 64)**		**<0.001**
Physical Functioning	87.36± 2.31	76.39 ± 2.52	0.002	
Bodily pain	83.38 ± 2.53	70.47 ± 2.75	0.001	
				**<0.001**
Physical component summary scale	79.68±1.6	72.07±1.74	0.002	
**Sub-groups (Marital status)**				
**PCOS**				**0.087**
**Controls**	**Single (n = 33)**	**Married (n = 107)**		**<0.001**
Physical Functioning	88.29±2.99	79.34±2.12	0.016	
Role limitation due to physical problems	85.17±4.05	71.73±2.88	0.008	
Social Functioning	84.23±2.67	77.03±1.9	0.03	
Bodily pain	87.55±3.18	72.39±2.26	<0.001	
Physical component summary scale	82.76±1.99	72.88±1.42	<0.001	**<0.001**
**Sub-groups (Parity)**				
**PCOS**				**0.06**
**Controls**	**Nulliparous (n = 69)**	**Parity ≥ 1 (n = 71)**		**0.013**
Physical Functioning	88.11±2.42	76.74±2.39	0.001	
Bodily pain	82.1±2.71	73±2.67	0.018	
Physical component summary scale	79.91±1.68	72.59±1.66	0.002	
**Sub-groups (Hirsutism)**				
**PCOS**	**Non hirsute (n = 88)**	**Hirsute (n = 54)**		**<0.001**
Vitality	55.88±2.15	46.15±2.74	0.006	
Social Functioning	78.04±3.09	59.25±3.95	<0.001	
Mental health	60.73±2.61	45.57±3.33	<0.001	
Mental component summary scale	65.27±1.99	54.63±2.54	0.001	**0.002**
**Sub-groups (BMI)**				
	**BMI < 25kg/m**^**2**^ **(n = 58)**	**BMI ≥ 25kg/m**^**2**^ **(n = 84)**	**Mancova P-value**	
**PCOS**				**<0.001**
Bodily pain	79.32 ±3.56	64.22 ±2.95	0.002	
Physical Functioning	88.86 ±3.01	74.37±2.49	<0.001	
Physical component summary scale	76.15 ± 2.52	68.03±2.08	0.01	**0.007**
Mental component summary scale	61.53±2.48	61.01±2.05	0.87	
**Controls**	**BMI < 25kg/m**^**2**^ **(n = 60)**	**BMI ≥ 25kg/m**^**2**^ **(n = 80)**		
Bodily pain	87.48±2.44	75.2±2.48	<0.001	**<0.001**
Physical component summary scale	79.38±1.66	72.93±1.69	0.01	**0.02**
Mental component summary scale	71.39±1.99	70.67±2.02	0.8	

*MANCOVA adjusted for age, parity, FG scores, period regularity

** Multivariate test

One-way Mancova analysis demonstrated significant effects of BMI categories on SF 36 scores after adjustment for age, parity, FG scores and period regularity (PCOS: Wilk’s λ = 0.81, F = 3.71, P< 0.001; non PCOS: Wilk’s λ = 0.77, F = 4.77, P<0.001); BP and PF domains of the SF 36 in cases and BP per se in controls were significantly higher in normal weight women than in overweight or obese ones (P<0.05). To evaluate the group differences in PCS and MCS, one-way Mancova was conducted. The multivariate effects for the two BMI categories were significant (PCOS: Wilk’s λ = 0.92, F = 5.2, P = 0.007; non PCOS: Wilk’s λ = 0.94, F = 4, P = 0.02). In both groups, the univariate analysis showed that the normal weight women had significantly higher PCS scores than overweight or obese ones (P< 0.05) (Table 4).

In cases, a significant correlation was seen between BMI and PF, r = -0.301, P<0.001; BP, r = -0.23, P = 0.006 and PCS r = -0.3, P = 0.007. In controls, significant correlation was seen between BMI and BP, r = - 0.3, P<0.001 and PCS r = -0.27, P<0.001(data in S7 Table). The differences between PF correlations in the two groups were statistically significant using Fisher’s Z test (Z = -2.41, P = 0.008).

## Discussion

This study showed that women with PCOS, compared to their age-BMI matched controls, had significantly lower scores, assessed by SF36, in five domains of health related quality of life, including vitality (VT), mental health (MH), social functioning (SF), bodily pain (BP) and general health perception (GH). We found that being normal weight was related with higher scores for physical functioning (PF) and bodily pain (BP) and physical component summary scale (PCS) in PCOS and for BP and PCS in the controls. The correlation between PF with BMI in women with PCOS was statistically significant different from those observed in controls; the negative impact of excess weight on physical domains of HRQOL in women with PCOS is stronger than in controls.

In the present study, women with PCOS had lower scores in five domains of health related quality of life, of which 3 domains (vitality, mental health, social functioning) were related to mental aspect of health related quality of life; it seems that, similar to results of previous studies, the mental health domain of SF 36 was affected more than the physical domain [21, 22]. Since socio-demographic factors among women with PCOS and controls were comparable; it could be concluded that features associated with PCOS result in decrease in the HRQOL of these women. Results of the current study demonstrated that consequences of excess weight on the physical aspects of HRQOL varied between women with PCOS and healthy controls, results in agreement with those of a study that reported comorbidity of obesity had a greater impact on HRQOL [23].

However our results differ from studies conducted mostly in western countries, showing a positive association between excess weight and reduction in the mental aspects of HRQOL [13, 15]. Given that cultural and social circumstances constitute the concept of ideal body weight [24], the differences observed in our results could be explained by the variations between Eastern and Western cultures. In some Eastern countries, being overweight is regarded as a manifestation of beauty or affluence; in the Chinese traditional culture, moderate overweight is always related to wealth, prosperity and happiness, as they believe ‘Liberal mind brings fat body’ [25], however other than this, Chinese culture is very different from Iranian culture.

Our data demonstrated that the HRQOL of Iranian women with PCOS is not influenced by demographic factors, except for age. A study of patients afflicted by chronic diseases, reported that demographic factors had no effect on their HRQOL [26]. Our data shows increasing age had a negative impact on PF and positive influence on MH among women with PCOS. The logical explanation for lower scores of PF in older women with PCOS is that increasing age causes a decline, in daily levels of physical activity. With increasing age, a linear decrement in physical activity level was documented [27]. The higher scores of MH in older women with PCOS may be explained by improved cycle regularity and more adaptation with PCOS features in aging women with PCOS [28]; however, in healthy controls, consistent with results of previous studies, demographic factors were reported to affect HRQOL [29]. In the control group, increasing age negatively impacted PF, BP and PCS, results not consistent with those of Montazeri who concluded that mean scores of all domains of SF 36, were diminished with increasing age [19]. These discrepancies may be due to differences in the study population; in Montazeri’s study, the age range of participants ranged between 15 to 65 years. Our data are however in line with other studies that showed older age and lower education to be associated with lower scores of the physical components of HRQOL [30]. Poorer HRQOL in less educated women may be due to their lower socio-economic status and limited access to health services [31]. However, our results differ with those of some studies in which compared to unmarried women, married ones had better HRQOL [30]; in our study, compared with married women, unmarried women had better HRQOL. Some studies confirm that marriage and exhausting housework caused a decrease, especially in the physical domains of HRQOL [29].

Our study, similar to others, demonstrated that scores of hirsutism were negatively associated with the emotion domain [32]. It has been shown that the clinical signs of hyperandrogenemia may cause a significant amount of emotional distress and lower self-esteem in women with PCOS [33], data also shows that hirsutism has a great role in women's identity; women’s feelings of femininity and attractiveness are noticeably reduced by hirsutism [5].

To the best of our knowledge, this study is the first to compare the association between BMI and HRQOL in women with PCOS and age and BMI matched controls. Anthropometric measurements and clinical assessment of hirsutism were both done by a single person (F.S). Using MANCOVA enabled us to adjust the results for all possible confounders.

The present study does have its limitations. We did not have adequate power for subgroup analysis of various PCOS phenotypes. In this study, a generic HRQOL instrument was used, whereas it was stated that obesity specific tools (the impact of weight on QOL questionnaire, health state preference assessment) [12] and specific questionnaires for assessment of health related quality of life of women with PCOS [34] may be more sensitive than generic ones. Since excess weight affects various aspects of HRQOL, the use of the SF36, a widely generic instrument, seems justifiable. Since, this was a case control study; we cannot conclude causality between excess weight and HRQOL. We did not measure androgen or use ultrasonography for selected controls to identify eumenorrheic non hirsute women, who may have less severe forms of PCOS.

## ‍Conclusion

While excess body weight adversely affected physical aspect of HRQOL in both PCOS and controls, this adverse effect is more prominent in women with PCOS.

## Supporting Information

S1 Data SPSS data file containing variables used in analysis. (SAV)

S1 Table The effect of age levels on HRQOL in women with or without PCOS. (DOC)

S2 Table The effect of marital status on HRQOL in women with or without PCOS. (DOC)

S3 Table The effect of educational levels on HRQOL in women with or without PCOS. (DOC)

S4 Table The effect of hirsutism on HRQOL in women with PCOS. (DOC)

S5 Table The effect of BMI on health related quality of life after adjusting for age, parity, FG scores and period regularity. (DOC)

S6 Table The effect of BMI on health related quality of life after adjusting for age, parity and FG scores. (DOC)

S7 Table Correlations (r) of BMI and domains of SF 36. (DOC)
