# Supplementary material for: Comparison of the Association of Excess Weight on Health Related Quality of Life of Women with Polycystic Ovary Syndrome: An Age- and BMI-Matched Case Control Study
Source: PLoS One. 2016 Oct 13;11(10):e0162911. doi: 10.1371/journal.pone.0162911 (PMC5063389; doi:10.1371/journal.pone.0162911)
Supplement: S1 Table — (DOC) [file pone.0162911.s002.doc]

**S1 Table: The effect of age levels on HRQOL in women with or without PCOS**

| **Cases** | **<30 years(n=79)** | **≥ 30 years(n=63)** | **MANOVA**  **P-value** | **Wilks' Lambda**  **P-value** |
| --- | --- | --- | --- | --- |
|  |  |  |  | **0.033** |
| Physical Functioning | 84.61±2.66 | 74.88±2.98 | 0.016 |  |
| Mental health | 50.48±2.82 | 60.65±3.16 | 0.018 |  |
| Role limitation due to physical problems | 76.37±4.08 | 65.76±4.57 | 0.08 |  |
| Bodily pain | 69.81±3.1 | 71.12±3.47 | 0.77 |  |
| GH | 63±2.43 | 63.09±2.72 | 0.98 |  |
| Role limitation due to emotional problems | 70.56±3.78 | 62.17±4.23 | 0.14 |  |
| Vitality | 50.67±2.32 | 54.07±2.6 | 0.33 |  |
| Social Functioning | 72.15±3.4 | 69.32±3.83 | 0.58 |  |
|  |  |  |  | **0.1** |
| PSC | 73.45±2.16 | 68.71±2.42 | 0.85 |  |
| MSC | 60.96±2.18 | 61.55±2.44 | 0.14 |  |
| **Controls** | **<30 years(n=76)** | **≥ 30 years(n=64)** | **MANOVA**  **P-value** | **Wilks' Lambda****  **P-value** |
|  |  |  |  | **<0.001** |
| Physical Functioning | 87.36±2.31 | 76.39±2.52 | 0.002 |  |
| Bodily pain | 83.38±2.53 | 70.47±2.75 | 0.001 |  |
| Role limitation due to physical problems | 78.42±3.25 | 73.66±3.54 | 0.32 |  |
| GH | 69.55±1.7 | 67.75±1.85 | 0.47 |  |
| Role limitation due to emotional problems | 70.3±3.85 | 72.42±4.2 | 0.71 |  |
| Vitality | 64.66 ±2.21 | 64.53±2.37 | 0.96 |  |
| Social Functioning | 76.42±2.1 | 77±2.29 | 0.8 |  |
| Mental health | 68.68±2.33 | 68.99±2.53 | 0.92 |  |
|  |  |  |  | **<0.001** |
| PCS | 79.68±1.6 | 72.07±1.74 | 0.002 |  |
| MSC | 70.01±1.83 | 72.25±1.99 | 0.41 |  |

* Multivariate test
