# Supplementary material for: Comparison of the Association of Excess Weight on Health Related Quality of Life of Women with Polycystic Ovary Syndrome: An Age- and BMI-Matched Case Control Study
Source: PLoS One. 2016 Oct 13;11(10):e0162911. doi: 10.1371/journal.pone.0162911 (PMC5063389; doi:10.1371/journal.pone.0162911)
Supplement: S2 Table — (DOC) [file pone.0162911.s003.doc]

**S2 Table: The effect of marital status on HRQOL in women with or without PCOS**

| **Cases** | **Single(n=32)** | **Married(n= 110)** | **MANOVA**  **P-value** | **Wilks' Lambda***  **P-value** |
| --- | --- | --- | --- | --- |
|  |  |  |  | **0.087** |
| Physical Functioning | 86.71±4.23 | 78.42±2.28 | 0.08 |  |
| Role limitation due to physical problems | 69.53±6.48 | 72.28±3.49 | 0.7 |  |
| Social Functioning | 62.5±5.32 | 73.34±2.87 | 0.07 |  |
| Bodily pain | 67.07±4.86 | 71.35±2.62 | 0.6 |  |
| GH | 59.84±3.81 | 63.97±2.05 | 0.34 |  |
| Role limitation due to emotional problems | 62.53±5.97 | 68.09±3.22 | 0.41 |  |
| Vitality | 50.94±3.66 | 52.54±1.97 | 0.7 |  |
| Mental health | 48.71±4.48 | 56.81±2.21 | 0.11 |  |
|  |  |  |  | **0.11** |
| PCS | 70.79±3.42 | 71.52±1.84 | 0.85 |  |
| MSC | 56.17±3.4 | 62.7±1.83 | 0.09 |  |
| **Controls** | **Single(n=33)** | **Married(n= 107)** | **MANOVA**  **P-value** | **Wilks' Lambda***  **P-value** |
|  |  |  |  | **<0.001** |
| Physical Functioning | 88.29±2.99 | 79.34±2.12 | 0.016 |  |
| Role limitation due to physical problems | 85.17±4.05 | 71.73±2.88 | 0.008 |  |
| Social Functioning | 84.23±2.67 | 77.03±1.9 | 0.03 |  |
| Bodily pain | 87.55±3.18 | 72.39±2.26 | <0.001 |  |
| GH | 70.03±2.16 | 68.07±1.53 | 0.46 |  |
| Role limitation due to emotional problems | 70.05±4.9 | 71.88±3.48 | 0.76 |  |
| Vitality | 64.62±2.76 | 64.58±1.96 | 0.99 |  |
| Mental health | 69.51±2.96 | 68.48±2.1 | 0.77 |  |
|  |  |  |  | **<0.001** |
| PCS | 82.76±1.99 | 72.88±1.42 | <0.001 |  |
| MSC | 72.1±2.33 | 70.49±1.65 | 0.57 |  |

* Multivariate test
