# Supplementary material for: Comparison of the Association of Excess Weight on Health Related Quality of Life of Women with Polycystic Ovary Syndrome: An Age- and BMI-Matched Case Control Study
Source: PLoS One. 2016 Oct 13;11(10):e0162911. doi: 10.1371/journal.pone.0162911 (PMC5063389; doi:10.1371/journal.pone.0162911)
Supplement: S3 Table — (DOC) [file pone.0162911.s004.doc]

**S3 Table: The effect of educational levels on HRQOL in women with or without PCOS**

| **Cases** | **Less than Diploma(n=22)** | **Diploma& higher(n=120)** | **MANOVA**  **P-value** | **Wilks' Lambda***  **P-value** |
| --- | --- | --- | --- | --- |
|  |  |  |  | **0.23** |
| Physical Functioning | 84.96±5.14 | 79.44±2.02 | 0.32 |  |
| Role limitation due to physical problems | 77.27±7. 8 | 70.63±3.34 | 0.43 |  |
| Social Functioning | 79.81±6.41 | 68.89±2.74 | 0.07 |  |
| Bodily pain | 64.79±5.85 | 71.42±2.5 | 0.3 |  |
| GH | 69.09±4.58 | 61.93±1.96 | 0.15 |  |
| Role limitation due to emotional problems | 77.25±7.16 | 64.93±3.06 | 0.11 |  |
| Vitality | 50.45±4.41 | 52.5±1.89 | 0.67 |  |
| Mental health | 54.22±5.45 | 55.13±2.33 | 0.87 |  |
|  |  |  |  | **0.11** |
| PCS | 74.03±4.11 | 70.85±1.76 | 0.48 |  |
| MSC | 65.93±4.12 | 60.36±1.76 | 0.21 |  |
| **Controls** | **Less than Diploma(n=33)** | **Diploma& higher(n=107)** | **MANOVA**  **P-value** | **Wilks' Lambda***  **P-value** |
|  |  |  |  | **0.001** |
| Physical Functioning | 79.31±3.63 | 83.28±2.01 | 0.34 |  |
| Role limitation due to physical problems | 72.17±4.94 | 77.5±2.74 | 0.34 |  |
| Social Functioning | 78.03±3.24 | 79.89±1.8 | 0.61 |  |
| Bodily pain | 66.22±3.84 | 80.95±2. 3 | 0.006 |  |
| GH | 68.02±2.58 | 68.94±1.43 | 0.75 |  |
| Role limitation due to emotional problems | 76.76±5.82 | 69.57±3.23 | 0.28 |  |
| Vitality | 66.21±3.29 | 64.1±1.83 | 0.57 |  |
| Mental health | 64.68±3.51 | 70.1±1.95 | 0.18 |  |
|  |  |  |  | **0.01** |
| PCS | 71.43±2.47 | 77.67±1.37 | 0.029 |  |
| MSC | 71.42±2.78 | 70.9±1.54 | 0.87 |  |

* Multivariate test
