# Supplementary material for: Comparison of the Association of Excess Weight on Health Related Quality of Life of Women with Polycystic Ovary Syndrome: An Age- and BMI-Matched Case Control Study
Source: PLoS One. 2016 Oct 13;11(10):e0162911. doi: 10.1371/journal.pone.0162911 (PMC5063389; doi:10.1371/journal.pone.0162911)
Supplement: S4 Table — (DOC) [file pone.0162911.s005.doc]

**S4** Table: The effect of hirsutism on HRQOL in women with PCOS

| **Cases** | **Non hirsute (n=88)** | **Hirsute (n=54)** | **MANOVA**  **P-value** | **Wilks' Lambda***  **P-value** |
| --- | --- | --- | --- | --- |
|  |  |  |  | **< 0.001** |
| Physical Functioning | 83.15 ± 2.55 | 75.64 ± 3.25 | 0.07 |  |
| Role limitation due to physical problems | 73.5 ± 3.9 | 69.07 ± 4.98 | 0.5 |  |
| Social Functioning | 78.04 ± 3.09 | 59.25 ± 3.95 | < 0.001 |  |
| Bodily pain | 71.13 ± 2.93 | 69.18 ± 3.75 | 0.68 |  |
| GH | 63.26 ± 2.3 | 62.68 ± 2.94 | 0.87 |  |
| Role limitation due to emotional problems | 66.41 ± 3.61 | 67.54 ± 4.61 | 0.84 |  |
| Vitality | 55.88 ± 2.15 | 46.15 ± 2.74 | 0.006 |  |
| Mental health | 60.73 ± 2.61 | 45.57 ± 3.33 | < 0.001 |  |
|  |  |  |  | **0.002** |
| PCS | 73.7 ± 2.01 | 69.14 ±2.62 | 0.28 |  |
| MSC | 65.27 ± 1.99 | 54.63 ± 2.54 | 0.001 |  |
