# Supplementary material for: Comparison of the Association of Excess Weight on Health Related Quality of Life of Women with Polycystic Ovary Syndrome: An Age- and BMI-Matched Case Control Study
Source: PLoS One. 2016 Oct 13;11(10):e0162911. doi: 10.1371/journal.pone.0162911 (PMC5063389; doi:10.1371/journal.pone.0162911)
Supplement: S5 Table — (DOC) [file pone.0162911.s006.doc]

**S5 Table: The effect of BMI on health related quality of life after adjusting for age, parity, FG scores and period regularity**

| **Cases** | **BMI < 25kg/m^2^**  **(n= 58)** | **BMI ≥ 25kg/m^2^**  **(n=84)** | **MANCOVA***  **P-value** | **Wilks' Lambda****  **P-value** |
| --- | --- | --- | --- | --- |
|  |  |  |  | **<0.001** |
| Bodily pain | 79.32 ± 3.56 | 64.22 ± 2.95 | 0.002 |  |
| Physical Functioning | 88.86 ± 3.01 | 74.37 ± 2.49 | <0.001 |  |
| Role limitation due to physical problems | 75.21 ± 4.87 | 69.21 ± 4.03 | 0.35 |  |
| GH | 61.21 ± 2.88 | 64.3 ± 2.38 | 0.41 |  |
| Role limitation due to emotional problems | 64.11 ± 4.5 | 68.73 ± 3.72 | 0.43 |  |
| Vitality | 52.43 ± 2.65 | 52.01 ± 2.19 | 0.9 |  |
| Social functioning | 72.69 ± 3.92 | 69.65 ± 3.24 | 0.55 |  |
| Mental health | 56.9 ± 3.12 | 53.67 ± 2.58 | 0.43 |  |
|  |  |  |  | **0.007** |
| PCS | 76.15 ± 2.52 | 68. 03 ± 2.08 | 0.01 |  |
| MSC | 61.53 ± 2.48 | 61.01± 2.05 | 0.87 |  |

* MANCOVA adjusted for age, parity, FG scores and period regularity

** Multivariate test
