# Supplementary material for: Comparison of the Association of Excess Weight on Health Related Quality of Life of Women with Polycystic Ovary Syndrome: An Age- and BMI-Matched Case Control Study
Source: PLoS One. 2016 Oct 13;11(10):e0162911. doi: 10.1371/journal.pone.0162911 (PMC5063389; doi:10.1371/journal.pone.0162911)
Supplement: S6 Table — (DOC) [file pone.0162911.s007.doc]

**S6 Table: The effect of BMI on health related quality of life after adjusting for age, parity and FG scores**

| **Controls** | **BMI < 25kg/m^2^**  **(n= 71)** | **BMI ≥ 25kg/m^2^**  **(n=69)** | **MANCOVA***  **P-value** | **Wilks' Lambda****  **P-value**  **<0.001** |
| --- | --- | --- | --- | --- |
| Bodily pain | 87.48 ± 2.44 | 75.2 ± 2.48 | <0.001 |  |
| Physical Functioning | 80.91± 2. 4 | 83.83 ± 2.44 | 0.41 |  |
| Role limitation due to physical problems | 79.24 ± 3.5 | 73.16 ± 3.56 | 0. 24 |  |
| GH | 69.89 ± 1.83 | 67.52 ± 1.86 | 0.38 |  |
| Role limitation due to emotional problems | 70.15 ± 4.15 | 72.42 ± 4.22 | 0.71 |  |
| Vitality | 65.59 ± 2.35 | 63.57 ± 2.39 | 0.56 |  |
| Social Functioning | 78.66 ± 2.31 | 80.26 ±2.35 | 0.64 |  |
| Mental health | 71.15 ± 2.51 | 66.43 ± 2.55 | 0.2 |  |
|  |  |  |  | **0.02** |
| PCS | 79.38 ± 1.66 | 72.93 ± 1.69 | 0.01 |  |
| MSC | 71.39 ± 1.99 | 70.67 ± 2.02 | 0.8 |  |

* MANCOVA adjusted for age, parity and FG scores

** Multivariate test
