# Supplementary material for: Comparison of the Association of Excess Weight on Health Related Quality of Life of Women with Polycystic Ovary Syndrome: An Age- and BMI-Matched Case Control Study
Source: PLoS One. 2016 Oct 13;11(10):e0162911. doi: 10.1371/journal.pone.0162911 (PMC5063389; doi:10.1371/journal.pone.0162911)
Supplement: S7 Table — (DOC) [file pone.0162911.s008.doc]

**S7 Table: Correlations (r) of BMI and domains of SF 36**

| **SF- 36** | **Cases**  **(r)** | **P value before adjusting for age, parity, FG scores and period regularity** | **Cases**  **(r)** | **P value after adjusting for age, parity, FG scores and period regularity** |
| --- | --- | --- | --- | --- |
| Physical Function | -0.338 | P<0.001 | -0.301 | P<0.001 |
| Bodily pain | -0.441 | P=0.005 | -0.23 | P=0.006 |
| PCS | -0.248 | P=0.003 | -0.3 | P=0.007 |
| Role limitation due to physical problems | -0.152 | P=0.071 | -0.13 | P=0.12 |
| GH | -0.039 | P=0.64 | 0.04 | P=0.64 |
| Role limitation due to emotional problems | 0.031 | P=0.71 | -0.29 | P=0.73 |
| Vitality | 0.084 | P=0.32 | 0.09 | P=0.28 |
| Social Functioning | -0.010 | P=0.21 | -0.083 | P=0.33 |
| Mental health | -0.066 | P=0.43 | -0.079 | P=0.35 |
| MSC | -0.054 | P=0.52 | -0.047 | P=0.58 |
| **SF- 36** | **Controls**  **(r)** | **P value before adjusting for age, parity and FG scores** | **Controls**  **(r)** | **P value after adjusting for age, parity and FG scores** |
| Physical Function | -0.14 | P=0.043 | -0.014 | P=0.86 |
| Bodily pain | -0.236 | P<0.001 | -0.3 | P<0.001 |
| PCS | -0.362 | P<0.001 | -0.27 | P=0.001 |
| Role limitation due to physical problems | -0.196 | P=0.52 | -0.17 | P=0.39 |
| GH | -0.12 | P=0.18 | -0.1 | P=0.18 |
| Role limitation due to emotional problems | -0.02 | P=0.81 | -0.036 | P=0.67 |
| Vitality | -0.043 | P=0.61 | -0.026 | P=0.76 |
| Social Functioning | 0.021 | P=0.8 | -0.007 | P=0.93 |
| Mental health | -0.11 | P=0.11 | -0.1 | P=0.2 |
| MSC | -0.05 | P=0.54 | -0.06 | P=0.46 |
